# Medical uses for phenol in the urinary tract: A possible forgotten treatment (Review)

**DOI:** 10.3892/mi.2021.13

**Published:** 2021-08-31

**Authors:** Takuya Sadahira, Yuki Maruyama, Toyohiko Watanabe, Takanori Sekito, Yosuke Mitsui, Koichiro Wada, Motoo Araki, Masami Watanabe

**Affiliations:** 1 Department of Urology, Okayama University Graduate School of Medicine, Dentistry and Pharmaceutical Sciences, Okayama 700-8558, Japan; 2 Department of Urology, Shimane University School of Medicine, Izumo, Shimane 693-0021, Japan; 3 Center for Innovative Clinical Medicine, Okayama University Hospital, Okayama 700-8558, Japan

**Keywords:** phenol, urinary tract, urinary bladder, therapeutic

## Abstract

Phenol is a chemical compound that was first used medically as an antiseptic. At low concentrations, phenol exerts local anesthetic effects achieved through denervation; at high concentrations, it exerts a potent protein-denaturing effect that induces apoptosis. Phenol injection therapy has a long history of use in urology. It is reportedly effective for hemorrhagic cystitis, benign prostate hyperplasia, overactive bladder, hydrocele, bladder tumors, interstitial cystitis and other benign urologic diseases, and it is also used as a tool to decrease bleeding during prostate surgery. The present review article summarizes the medical applications of phenol in urological field. The articles available on the medical uses of phenol are primarily older and retrospective, involving small numbers of patients. In the absence of comparative studies with other treatments, it is impossible to determine the relative benefit of phenol. However, the treatment outcomes of phenol injection are fairly well-established. Phenol therapy may be an option for patients who are poor candidates for invasive treatment. Further studies are required, however, as are improvements in the injection technique to reduce the rate of complications.

## 1. Introduction

Joseph Lister first used phenol in a medical capacity in 1865, as an antiseptic to sterilize operating fields, with favorable results (1). In addition to its antimicrobial actions, phenol exerts local anesthetic effects at low concentrations. These effects are achieved through denervation (2,3). At high concentrations, phenol exerts a potent protein-denaturing effect (4). Due to its various effects, phenol is used in a wide range of fields (3-9). However, there are only a limited number of reviews available on the medical applications of phenol. In the present review, the medical applications of phenol in urological field are summarized.

## 2. Medical uses for phenol in the bladder

### Hemorrhagic cystitis (HC)

HC is a condition associated with certain disease states, as well as with exposure to drugs, viruses and toxins. Diffuse inflammation causes bleeding from the bladder mucosa (9). Management options include coagulation, embolization, fulguration, hyperbaric oxygen therapy and surgical treatment (10). However, some patients experience intractable HC that is difficult to manage, particularly when the condition develops after the use of cyclophosphamide chemotherapy. In such patients, the intravesical instillation of phenol is an effective, minimally invasive and less morbid option compared with conventional treatments (11). The intravesical instillation of phenol is reportedly effective for chemical cauterization in patients with severe bladder hemorrhage (11,12). In a clinical experiment, phenol was shown to destroy the urothelium, but not muscle (13). The bladder capacity diminishes temporarily and then returns to its normal size with the complete epithelialization of the urothelium. The treatment is associated with a low incidence of bladder fibrosis (13). A reported complication of phenol involved failure to drain the bladder following instillation. The infant suffered severe methemoglobinemia (14).

Previous case reports describe two children with leukemia, 12 and 16 years of age, who were administered phenol for refractory HC following cyclophosphamide therapy (14,15). The method requires suprapubic cystostomy under anesthesia. A total of 30 cc of 100% phenol with 30 cc of glycerin is instilled and left in the bladder for 1 min. The mixture is then removed by suction followed by the instillation of 60 cc absolute ethanol (90 or 95%) to neutralize the effect of any remaining phenol. After 1 min, the ethanol is suctioned out and the bladder is irrigated with copious amounts of saline. The bladder is closed, and a 30 F suprapubic Foley catheter is left in place (14,15). Both case reports describe the immediate post-operative clearing of the urine, and both patients had their suprapubic catheter removed 6 weeks after surgery (14,15). Initially, the patients had a small bladder capacity on intravenous pyelogram and were unable to feel the sensation of needing to void. Within 2-4 weeks, the patients recovered sensation, reasonable bladder control and adequate bladder capacity. Both of these reports describe the use of ethanol to wash out the remaining phenol in the bladder (14,15). Phenol does not dissolve in water at relatively high concentrations, although it does dissolve uniformly in water at low concentrations (≤7%) (16). Thus, it may be possible to omit the step of ethanol instillation.

Phenol is also effective when silver nitrate fails to control bleeding (17). The mucosa is swabbed with a 1:1 phenol-alcohol solution, and bleeding is immediately terminated. Duckett *et al* (15) described the successful control of HC by phenol cauterization in three patients, although the details of treatment are unclear.

Severe vesical bleeding in children is difficult to manage as the small urethra precludes the use of large catheters for bladder irrigation and clot removal. Thus, phenol may be particularly useful in pediatric patients (18). However, since experience with phenol is limited, further investigations are required to determine the usefulness of injection therapy.

### Overactive bladder

Subtrigonal phenol injection has been employed since 1982 for patients with detrusor instability, detrusor hyperreflexia and bladder hypersensitivity who have not had success with more conservative treatments (19). Ewing *et al* (19) were the first to describe this technique in 1982. Cystoscopy is performed under general anesthesia, and a 35-cm, 20-gauge Shuttleworth needle is passed through the bladder wall and advanced to midway between the bladder neck and the ureteric office on each side. A total of 10 ml 6% phenol solution is injected into each side through transvaginal approach. An indwelling Foley catheter is left overnight and removed the following morning. The overall response rate in the 30 patients in the study by Ewing *et al* (19) was 62.5% at 1 year. In subsequent studies, the results of treatment in the early post-injection period ranged from 29 to 63% (20-25). Blackford *et al* reported the highest number of patients: 62 of their 116 patients (53%) had a symptomatic response at 3 months after the phenol injection (25). A satisfactory response rate was more likely in patients with detrusor hyperreflexia (82%), older patients with detrusor instability (69%) and patients with idiopathic bladder hypersensitivity (68%), but less likely in younger patients with detrusor instability (14%) and in patients who had bladder hypersensitivity with a definable cause, such as interstitial cystitis (0%) (25). Other researchers have noted a diminishing effect of infusion therapy over time. Rosenbaum *et al* (23) reported that the overall success rate at 1 month was 48%, decreasing to 16% at 6 months and to 3% at 1 year. Chapple *et al* (21) also reported that 10 of their 18 female patients exhibited an improvement in their condition at 1 month; however, this figure decreased to 7 out of 18 at 3 months and to 2 out of 18 at 6 months. These results demonstrate that clinical denervation following a transtrigonal phenol injection with a 6% phenol aqueous solution may be, at best, only transient.

Some concerning complications are sometimes observed after the injection. Ewing *et al* (19) reported that 5 of their 30 patients experienced chronic urinary retention, and 2 patients experienced a trigonal ulcer and transient ureteric reflux; the ulcer and the reflux resolved spontaneously. Mclnerney *et al* (20) also described a significant complication rate (17%) for phenol injection. The most common complication in their study was urinary retention, occurring in 8 of 72 patients (20). In addition, 4 patients experienced nerve palsies, and 2 patients developed localized bladder mucosal necrosis at the injection site; one of these patients experienced calculus formation. One of the 9 male patients developed erectile dysfunction (20). The authors of other series have reported fistula formation, significant hematuria and ureteric stenosis (21-25). Vesicovaginal fistula is a particularly severe complication that can arise from placing phenol too medially. Extreme caution should be exercised when a second injection is necessary, e.g., in patients with an inadequate response to an initial injection.

These findings suggest that the use of transtrigonal phenol for the treatment of overactive bladder is difficult to justify using the reported methods. One reason for the poor results and the resultant complications may be that phenol diffuses widely through the perivesical fat (23). Improving the injection technique to prevent phenol diffusion could lead to greater efficacy and may prevent complications. Further research and development in this area is required.

### Bladder tumors

Multiple papillomatosis of the bladder and diffuse non-infiltrating papillary carcinoma of the bladder are conditions that are difficult to assess using transurethral techniques. Unless each individual lesion is removed and staged, the variation in malignant potential among lesions cannot be accurately assessed. Vermooten *et al* (26) overcame this difficulty by using phenol to induce complete destruction of the bladder mucosa in the hope that the newly regenerated epithelium would not show a neoplastic tendency. Modifying a suggestion of Kirwin (27), they reported the successful use of phenol for chemical cauterization of bladder mucosal tumors in 13 patients with papillomatosis (26), using a solution consisting of equal parts pure phenol and glycerin. The bladder capacity was initially diminished but later returned to an adequate size. Treatment appears to be efficient in ridding the bladder mucosa of papillary lesions, and recurrences are infrequent following the complete destruction of the bladder mucosa, though they do occasionally occur. Filling the bladder with phenol does not appear to be associated with treatment-related mortality.

To investigate how deeply phenol penetrates and the time required for regeneration of the bladder epithelium, Vermooten *et al* (26) performed experiments using canines. Under general anesthesia, the bladder was filled with 150 cc of a solution of equal parts pure phenol and glycerin; the solution was left in place for 2 min. The mixture was then removed and the bladder was washed with 95% alcohol. The animals were serially sacrificed. At 5 days, the intravesical ureter and ureteral orifice were noted to have an intact epithelium with a fibropurulent eschar replacing the destroyed bladder epithelium. Edema and infiltration did not extend into the muscular layer. At 7 days, the epithelium had begun to regenerate from the ureteral orifices, replacing the eschar on the mucosal surface. At 21 days, the bladder mucosal epithelium had regenerated to its normal thickness, inflammation and edema had diminished, and there was no evidence of dysplasia or neoplasia. Cystography revealed no evidence of reflux. Phenol injection for chemical cauterization of the bladder mucosa for papillomatosis appears to be safe, based on this animal model (26).

### Interstitial cystitis

Interstitial cystitis is a disease of unknown etiology in which the main symptoms are bladder pain, urinary frequency and urgency (28). Particularly in the presence of Hunner's ulcers, it is associated with severe, recurrent bladder pain. The glycosaminoglycan layer of the bladder mucosa is impaired in patients with interstitial cystitis, and sensory nerves, such as the unmyelinated C-fibers are stimulated by urine, causing pain. A number of types of treatment have been advocated (29,30), including denervation using local application of phenol in the bladder. Investigators have applied various strengths of phenol solution directly to the ulcer (31,32); the use of 50% phenol and pure phenol are reportedly effective (29). The application of phenol causes protein coagulation, which leads to non-selective tissue destruction and the initiation of degeneration in nerve fibers, with a neurolytic effect that lasts for several months (32). However, neurodestructive modalities, including phenol, have potential complication and adverse effects. Advances in phenol injection techniques are required to solve these issues. The authors of the present review previously developed a novel, *in situ* permeation system that controlled the intratissue diffusion of therapeutic agents (33). This system will enable the localized ablation of target lesions.

## 3. Medical uses for phenol in the prostate

### Injection into the prostate

Phenol injection has also been used in the treatment of diseases of the prostate (34-36). The origin of intraprostatic phenol injection therapy was described by Talwar and Pande (37) in 1966, who treated 188 patients with benign prostate hyperplasia (BPH) with a 78.2% success rate. They used a composition of 2% liquid phenol, 2% glacial acetic acid, and 4% glycerin in distilled water. The patient was placed in the left lateral position and the prostate was injected with 2-3 ml phenol solution through a lumbar puncture needle. The needle was inserted into the perineum and guided into the gland using a finger in the rectum. An indwelling catheter was left in place and removed within 5-7 days. The injection was repeated if micturition was not restored in patients with urinary retention. If the BPH of the patient is of an early stage and the patient is not suffering from urinary retention, the injection can be performed as an outpatient procedure (37). Following this first description, there have been several reports of phenol injection for BPH in patients with or without urinary retention (34,38-40). The success rate varies from 56.4%, reported by Sharma and Goel (34), to 100%, reported by Shipman and Akile (39).

Intraprostatic phenol injection therapy is based on the concept of chemical necrosis and the consequent sloughing or shrinkage of obstructive tissue. In a study on 13 patients who received injection therapy, the mean estimated prostate size decreased from 31 g pre-operatively to 21 g post-operatively, and the residual urine volume was significantly improved (41). Complications are relatively rare and mild; these include hemorrhage, pain, impotence and poor urinary control. Perineal pain is the most common complication, occurring within a few minutes of injection and lasting up to several days (38,41). Plain pelvic radiographs obtained after the injection indicate that perineal pain may be associated with the extravasation of the solution outside the prostatic capsule (41).

This technique may be particularly useful for select patients who are at risk of poor outcomes following surgical treatment. However, the exact efficacy of injection therapy remains unclear as it is not uncommon to observed the spontaneous restoration of the ability to void following a period of catheter drainage of the bladder, and symptoms may improve following placebo treatment (42,43).

The intraprostatic injection of phenol solution has been adapted to minimize blood loss during prostatectomy or the transurethral resection of the prostate (TURP) (35,36). In open radical prostatectomy, a transperineal injection of 2 ml of 5% phenol in an almond oil solution is injected into the prostate at 48 h prior to surgery. A prospective study on 100 patients undergoing prostatectomy found that none of the 50 patients who received a phenol injection experienced perineal pain (35). In these patients, blood loss ranged from 20-120 ml, while blood loss in the non-injected group ranged from 175-680 ml. None of the injected patients required blood transfusion post-operatively, whereas six of the non-injected patients required transfusion for excessive blood loss or low hemoglobin values. Coagulative necrosis produced by the sclerosant phenol solution seems to reduce vascularity and renders it technically easier to enucleate the prostate, thereby reducing operative time (35).

In 1991, Szewczyk (36) used a 5% phenol solution in almond oil for patients undergoing TURP. The volume of resected prostate tissue, operative time and blood loss were compared among three groups: Those who were administered an injection of phenol solution 2 days prior to TURP, those who were administered the injection 5 days prior to TURP and a non-injected group. No differences were observed between the groups for any of the three factors (36). It is possible that 5 days is an inadequate time frame to produce the necessary necrosis of prostatic tissue to diminish surgical bleeding. Currently, laparoscopic surgery, robotic surgery and holmium laser nucleation are the mainstays of treatment for prostate disease; the utility of phenol injection for these procedures is unclear.

Complications due to phenol injection are pain towards the tip of the penis, a temperature >38˚C persisting <48 h after the injection, pain in the perineum and hematuria. There have never been any severe consequences or systemic side-effects reported due to phenol injection (35-43), at least to the best of our knowledge. However, cases of latent and occult prostate cancer may have not been noted and the injection may have increased the chance of metastases. In these studies, the injection was administered when prostate cancer had been excluded clinically.

## 4. Uses of phenol in other diseases

### Hydrocele

Sclerotherapy using phenol has been proposed as an alternative therapy to surgical treatment for patients with hydrocele (44,45). Moloney (44) first reported the use of injection therapy in 1975, using a 21% phenol solution. The treatment requires one to three outpatient visits, has an almost negligible complication rate and has had no treatment failures. Other investigators have used low concentrations of phenol: The cure rate after a single injection was reportedly 48 to 52% using 2.5% phenol, 58% using 3% phenol and 70.5% using 5% phenol (45-47). East and DuQuesnay (46) suggested that 5% phenol yielded significantly superior results than a 2.5% phenol injection (P=0.002 and P=0.012), and a 100% cure rate was achieved following three to four instillations. As sclerotherapy has fewer complications, a lower morbidity and is more cost-effective than other treatments, a number of chemicals in addition to phenol have been investigated, including tetracycline, sodium tetradecyl sulfate, polidocanol, fibrin glue, picibanil (OK-432), ethanolamine oleate, antazoline, rifampicin and talc (48). Although the use of any of these treatments by injection may readily be performed in a consulting room, repeat treatment is sometimes required, the efficacy compared with surgery is controversial, and patient satisfaction is lower than that with surgery. Thus, injection therapy is not currently considered a standard treatment for hydrocele.

### Sympathetic block for pain

The local anesthetic effects that phenol achieves through denervation have been applied to the use of sympathetic block for chronic prostatic pain. Patients with chronic prostatitis in whom conventional chemotherapy and repeated transurethral resection have failed to relieve pain are able to achieve complete relief with a permanent sympathetic block using phenol (49). Pain from the prostate gland can be mediated either via parasympathetic fibers from the caudal portion of the pelvic plexus or via sympathetic chain fibers from the lumbar segments of the spinal cord. Chemical sympathectomy using phenol (3 ml 7% phenol in water) is performed at L4, first on the left side and 4 days later on the right side. The needle position is carefully examined radiologically using both anteroposterior and lateral views. Complete pain relief is achieved without complication (49).

## 5. Conclusion and future perspectives

The therapeutic application of phenol is reportedly effective for a wide variety of urologic diseases (Table I). However, reports describing this therapy are primarily older and rely on small cohorts of patients. Currently, this treatment is not listed in the guidelines due to its complications. In additions, phenol, which is an environmental pollutant, and can cause acute renal failure, may require a more impartial and more careful interpretation and analysis. By changing the method of administration (e.g., local ablation), it is considered that highly effective drugs, such as phenol, which have been considered as deleterious drugs, can be used safely and may become a treatment option for refractory urinary tract diseases. Medical technologies are advancing daily, with techniques such as robotic surgery and laser therapy coming to the fore. The interest in minimally invasive treatment is increasing, and phenol offers the advantages of avoiding unnecessary hospitalization and surgery. However, in order for phenol to be used more effectively with fewer complications, it is necessary to improve the therapeutic techniques. In the future, phenol may play a role in the treatment of urinary tract diseases as an active medical agent.

## Figures and Tables

**Table I:** Summary of the applications of phenol in diseases.

Disease/condition	Composition	Injection	After injection	Major complication	(Refs.)
Hemorrhagic cystitis	Phenol 100% 30 cc + glycerin 30 cc	Intravesical (suprapubic cystostomy)	Washed with 90 or 95% ethanol (60 cc)	Frequency of urination within 4 weeks	(14,15)
Overactive bladder	10 ml of 6% aqueous phenol into each side	Subtrigonal (35 cm, 20 G Shuttleworth needle)	Non-washing	Diminishing effect Urinary retention Nerve palsies	(19-25)
Bladder tumors	Phenol 100% 75 cc + glycerin 75 cc	Intravesical	Washed with 95% alcohol	Bladder capacity initially diminished	(26,27)
Interstitial cystitis	Phenol 100 or 50%	Intravesical	Not described	Not described	(29-32)
Prostate					
BPH	2% Phenol, 2% glacial acetic acid, 4% glycerin in distilled water (2-3 cc)	Transperineal (left lateral position, lumber puncture needle)	Non-washing	Perineal pain Hemorrhage Impotence	(34-41)
Surgery (reduce blood loss)	5% Phenol in an almond oil solution	Transperineal	Non-washing	Not described	(35,36)
Hydrocele	2.5-5% Phenol in water (cc; depending amount of fluid evacuated)	Through scrotal wall into the hydrocele	Non-washing	Required repeat treatment	(44-48)
Pain	3 cc 7% Phenol in H_2_O	A sympathetic block at the level of L4	First on left side 4 days after right side	No complication reported	(49)

BPH, benign prostate hyperplasia.

## Data Availability

The datasets used and/or analyzed during the current study are available from the corresponding author on reasonable request.
